# Can thromboprophylaxis build a link for cancer patients undergoing surgical and/or chemotherapy treatment? The MeTHOS cohort study

**DOI:** 10.1007/s00520-022-07096-1

**Published:** 2022-05-12

**Authors:** Spyridon Xynogalos, David Simeonidis, George Papageorgiou, Abraham Pouliakis, Nikolaos Charalambakis, Evangelos Lianos, Evridiki Mazlimoglou, Alexandros-Nikolaos Liatsos, Christos Kosmas, Nicolaos Ziras

**Affiliations:** 1 Department of Medical Oncology, “METAXA” Memorial Piraeus Cancer Hospital, Mpotasi 51, 185 37 Piraeus, Greece; 2 2nd Department of Pathology, National and Kapodistrian University of Athens, University General Hospital “Attikon”, Athens, Greece

**Keywords:** Thrombosis, Cancer-associated thrombosis, Heparin, Low molecular weight heparin, LMWH, Tinzaparin, Venous thromboembolism, Pulmonary embolism, Bleeding, Conformance

## Abstract

**Background:**

Patients with active cancer have a 4–sevenfold increased risk for venous thromboembolism (VTE) especially during systematic anticancer treatment. Simultaneously, surgery is an additional risk factor.

**Methods:**

The Metaxas’s Hospital THromboprophylaxis program in Oncological & Surgical Patients (MeTHOS) is a prospective, phase IV, observational, non-interventional cohort study, aiming to record the thromboprophylaxis practice patterns in high-risk active cancer patients undergoing surgical and/or chemotherapy treatment.

**Results:**

We are reporting results from 291 ambulatory patients (median age: 67 years, Q1–Q3: 59–73 years, 54.6% males) who received anti-neoplastic treatment and administered thromboprophylaxis. 59.8% had cardiovascular disease (mostly hypertension), 76.6% were reported as having at least one comorbidity, while 27.5% and 15.8% accumulated two and three comorbidities, respectively. 94.9% of the patients were receiving highly thrombogenic agents such as platinum-based agents, 5-FU, immunotherapy, antiangiogenics/anti-VEGF, or erythropoietin. 26.5% of the patients were initially surgically treated. In terms of anticoagulation, all patients were treated with tinzaparin (fixed dose, 10,000 Anti-Xa IU, OD). The median anticoagulation duration was 6.2 months. Six thrombotic events were observed (2.06%, 95% CI: 0.76–4.43%): 5 were DVT, and one PE. With respect to safety, 7 bleeding events occurred (2.6%, 95% CI: 1.0–5.3%); 6 of them were minor.

**Conclusions:**

Thromboprophylaxis with LMWH in patients with active cancer and high thrombotic burden was safe and effective. Intermediate dose of tinzaparin seems to be an appropriate agent for cancer-associated thromboprophylaxis management.

**Clinical trial registration:**

ClinicalTrials.gov: NCT04248348.

## Introduction

Patients with active cancer have a 4–sevenfold increased risk of experiencing a venous thromboembolism (VTE) which is an independent risk factor for mortality, especially during the first 4 cycles of chemotherapy, in cancer patients of all stages [1, 2]. Furthermore, the incidence of cancer-associated thromboembolism (CAT) is increasing worldwide with the growing age and cancer prevalence, the enhanced detection of incidental thrombosis through CT scan, and the greater thrombogenicity of multiagent chemotherapeutic regimens [3, 4].

Additional risk factors for VTE include tumor type, stage and extent of the malignancy, as well as, the treatment with antineoplastic agents or surgery. Patient related factors, such as comorbidities and low degree of mobility can increase the thrombogenicity potential. Moreover, laboratory parameters (e.g., hemoglobin, platelets, and leukocytes) and other biomarkers (e.g., TF, TF-microparticles, thrombin, pro-inflammatory cytokines, soluble P-selectin, D-dimer and CRP) are predictive markers for the risk of VTE in cancer patients and have been used to enhance risk stratification [5, 6].

Besides the thrombotic risk, the bleeding risk for patients with active cancer needs also to be assessed [7]. Factors including age, platelet count, renal and liver status, invasive diagnostic/surgical procedures, recent immobility, recent bleeding, the cancer type and intracranial malignancy, metastasis, and systemic anticancer treatment should be taken into consideration [8]. Major challenge constitutes the management of thrombotic and bleeding risk in patients being under anticoagulation. Moreover, the bleeding risk from gastrointestinal (GI) tumors or genitourinary (GU) sites (e.g., nephrostomy tubes) should be taken into account when choosing anticoagulant agents [8].

The issue of drug-drug interactions (DDIs) is a major concern in the management of thrombosis in patients with active cancer and complicates further the selection of the proper treatment [9]. Leeuwen et al. reported that 46% of cancer patients were exposed to at least one DDI. Furthermore, 14% of these DDIs were life-threatening or exposing to permanent damage and 84% of these DDIs were exposing to a deterioration of patient’s status and a treatment was required, highlighting the clinical impact of DDIs in cancer [10].

A diligent reassessment prior to each cancer treatment line, along with the different anticancer agents administered, can facilitate the decision for thromboprophylaxis approach, and therefore, balance the various risks [11].

This thromboprophylaxis program (Metaxas’s Hospital THromboprophylaxis program in Oncological & Surgical Patients—MeTHOS, ClinicalTrials.gov identifier: NCT04248348) has been set in order to increase healthcare professionals’ awareness on the high thrombotic burden patient’s benefit. The program has provided the frame for collecting data for high thrombotic risk factors, thromboprophylaxis safety and efficacy, facilitating the awareness. Tinzaparin was chosen as an appropriate agent meeting the study needs.

## Materials and methods

### Study design

**Fig. 1:**
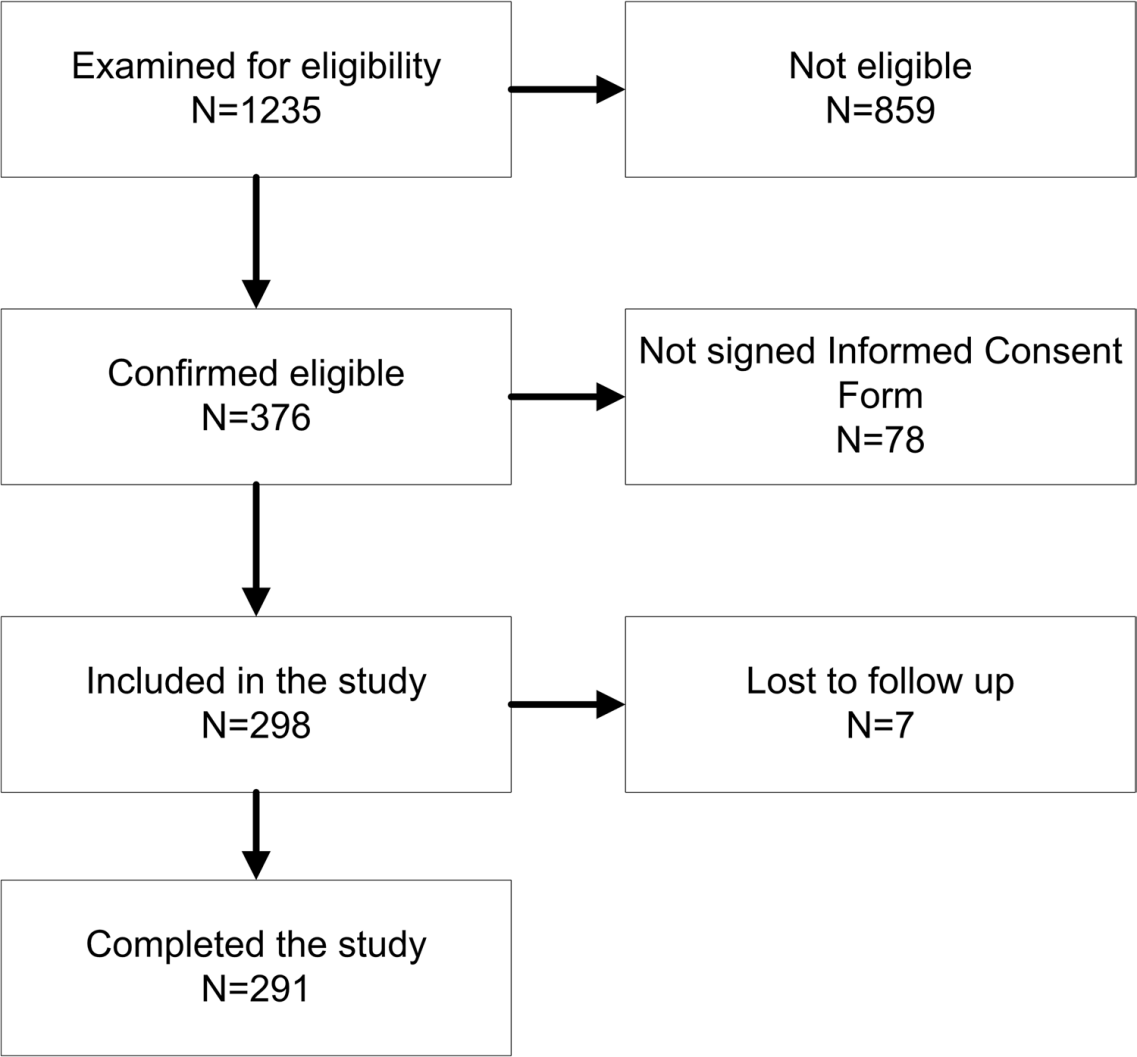
Flowchart of the study

The Metaxas’s Hospital THromboprophylaxis program in Oncological & Surgical Patients (MeTHOS, ClinicalTrials.gov identifier: NCT04248348) is a prospective, phase IV, observational, non-interventional cohort study, aiming to record the thromboprophylaxis practices in high-risk active cancer patients undergoing surgical and/or chemotherapy treatment. The inclusion criteria were as follows: (a) diagnosis of histological confirmed high thrombotic risk solid tumors (GI, thoracic, gynecologic, and genitourinary) undergoing surgery and/or chemotherapy, (b) age ≥ 18 years, (c) ECOG 0–2, (d) life expectancy > 6 months, (e) signed informed consent. Patients undergoing chemotherapy were managed with administration of tinzaparin (fixed dose of 0.5 ml, 10.000 Anti-Xa IU, OD). The study was conformant with Helsinki declaration and subsequent amendments and was approved by the bioethics committee of “METAXA” hospital (approval protocol number: 2394–5/2/2019). Each subject’s participation was designed to last from inclusion (enrolment visit) to the last follow-up visit by the end of systemic treatment and administration of thromboprophylaxis. The entire study was expected to last by the end of 2020. Since this was a single cohort observational study, no specific design for the number of patients was performed, instead all hospital patients meeting the enrolment criteria were eligible to participate. A flowchart showing the number of patients during each program stage is presented in Fig. 1.

Along with demographic and medical history data for each patient, cancer-related data, such as primary site, staging, metastasis status, patient performance status (PS) according to ECOG scale, anticancer and anticoagulation therapy information, surgical operation details and the use of central venous catheter, were also recorded. In relation to the main study outcomes, were recorded: (a) the number of thrombotic events, (b) the dose and duration of anti-thrombotic treatment, (c) any bleedings related to anticoagulation, and (d) patients’ adherence and compliance.

Thrombotic events were assessed by physical examination and subsequently by imaging methods [12, 13]. Bleeding events were categorized as follows: (a) major, (b) clinically relevant non-major bleeding, and (c) minor bleeding. Major bleedings were defined as clinically overt bleeding events associated with a fall in hemoglobin of 2.0 g/dL (1.24 mMol/L)  or more, or leading to a transfusion of ≥2 units of packed red blood cells or whole blood. As major bleedings were also defined bleedings in a critical area or organ such as: retroperitoneal, intracranial, intraocular, intraspinal, intra-articular, pericardial, and intramuscular with compartment syndrome. Additionally, a bleeding contributing to death was categorized as major bleeding. Clinically relevant non-major bleeding was defined as overt bleeding not meeting the criteria for major bleeding but associated with medical intervention, unscheduled contact (visit or telephone call) with a physician, (temporary) cessation of study treatment, or associated with discomfort for the patient such as pain, or impairment of activities of daily life. All other bleeding events were classified as minor. In cases of disagreement for the categorization of a bleeding event, an expert meeting was established in order to have a consensus and avoid any bias and additionally to attribute the bleeding to the disease or anticoagulation.

### Statistical analysis

The statistical analysis was performed within the environment of the R language software platform. Descriptive values were expressed as median and 1st–3rd quartile (Q1–Q3) range (as normality via the Kolmogorov–Smirnov was not assured) and for the categorical data using frequencies and the relevant percentages. Comparisons were made using the chi-square test (or the Fisher exact test for the cases of less than 5 expected cases in more than 25% of the contingency tale cells), and using nonparametric (Mann–Whitney *U*) tests for continuous variables. All tests were two sided and the significance level for all study variables was set *p* < 0.05. In cases of missing data, a case was either excluded from the study (if one of the primary outcomes was missing) or was used only in the part of analysis that the relevant data were available. During the first step of the statistical analysis, missing data were identified and efforts to collect them retrospectively were initiated. Moreover, patients lost to follow-up were not included; data processing as the evaluation of the primary outcomes was not possible.

## Results

In total, 291 ambulatory patients with active cancer receiving anticancer treatment were analyzed in the study. Their median age was 67 years and no difference (*p* = 0.3939) observed in the age of males and females. The median BMI of the study population was 26 kg/m^2^ with women having higher BMI (*p* = 0.0006). The characteristics of the study population are depicted in Table 1. Women had lower PS (*p* = 0.0100) than men. When counting comorbidities, 76.6% were reported as having at least one comorbidity, while 4, 3, or 2 comorbidities had 7.2%, 15.8%, and 27.5% of patients, respectively.

**Table 1:** Characteristics of the study population

	Characteristic	Valid cases*	Measure
	Patients	291	291
Patient related	Men (*n*, %)	291	159, 54.6%
	Age in years (median, Q1–Q3)	290	67, 59–73
	ΒΜΙ in kg/m^2^ (median, Q1–Q3)	289	26.0, 22.8–29.4
	Alcohol consumption	265	39.2%
	Smokers (ex or current)	274	67.2%
	Cardiovascular disease	277	59.8%
	Diabetes	276	31.9%
	Respiratory disease	276	32.3%
	Dyslipidemia	276	38.8%
	Thrombosis history VTE	291	15.5%
	Thrombosis history ATE	291	3.8%
	ECOG PS	291	
	0		26.8%
	1		46.1%
	2		26.1%
Treatment related	HTAs	291	97.9%
	Surgery	291	26.5%
	Central venous catheter	222	22.5%
Cancer related	Metastasis	291	72.9%
	Gastrointestinal system		37.1%
	Lung		22.7%
	Woman reproductive system		16.2%
	Breast		8.9%
	Urothelial system		8.3%
	Head and neck		2.4%
	Other (or unknown)		4.5%

*BMI* body mass index, *HTAs* highly thrombogenic agents, including erythropoietin

^*^Number of patents with available/valid data

**Fig. 2:**
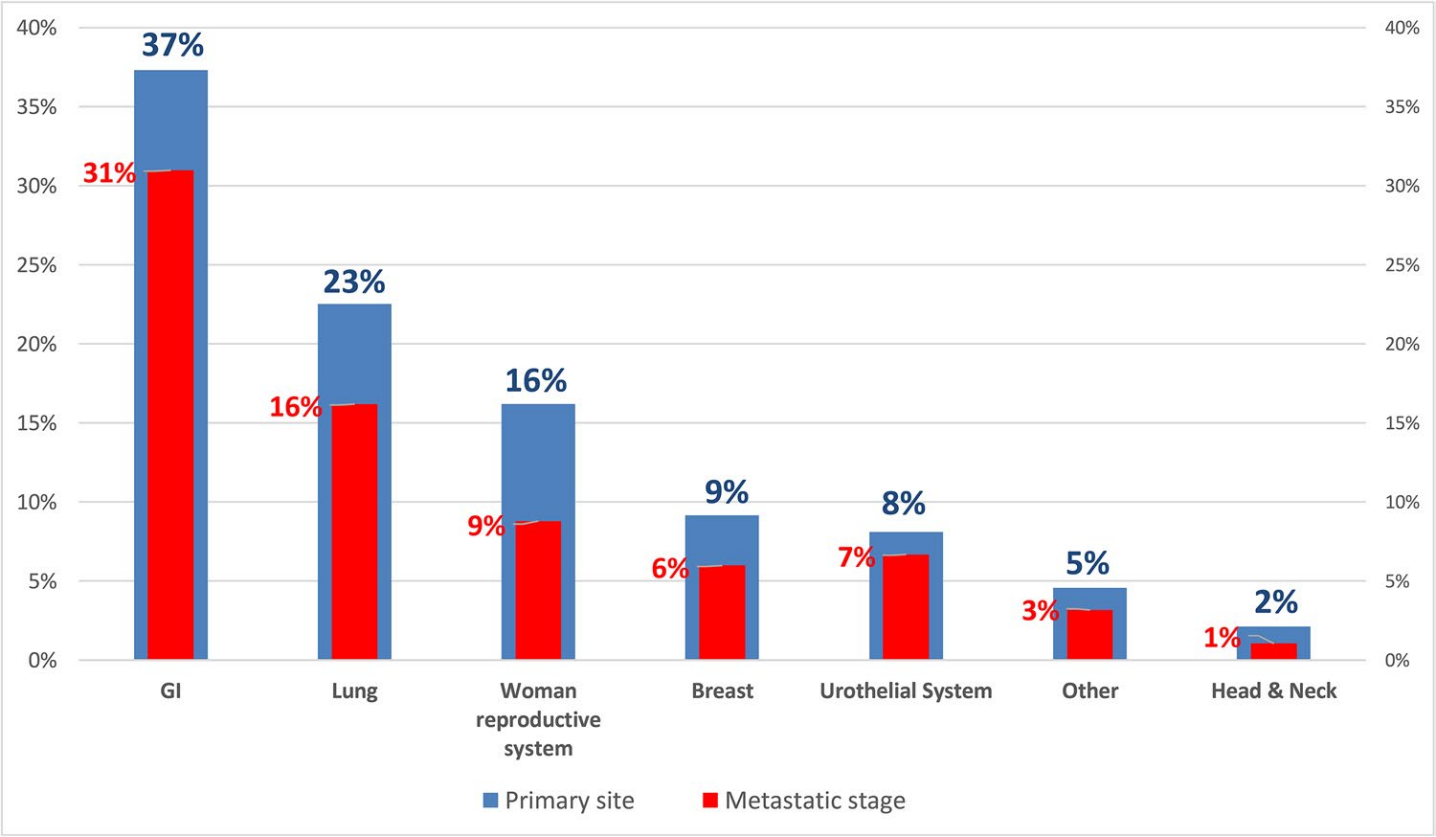
Cancer primary sites and metastatic disease. Metastasis bar (red) length is proportional to the percentage of metastasis within the primary site

There was a varying and highly thrombotic potential of the primary cancer sites, All patients included at this study had Khorana score ≥ 2; moreover, 108 (37.1%) cases involved the gastrointestinal system (among them: colorectal 36.1%, pancreas 33.3%, gastric 25%, and other sites of the GI system 5.6%), 66 cases (22.7%) involved the lung, 47 cases (16.2%) the woman reproductive system (ovaries 63.8%, uterine 29.8%, and cervix 6.4%), 26 (8.9%) the breast, 24 (8.3%) the urinary system (bladder 45.8%, prostate 33%, and renal 20.8%), 7 cases (2.4%) were head and neck cancers, and 13 (4.5%) cases had other (or unknown) primary site. Metastatic patients comprised 72.9% of the population (see Fig. 2 for a graphical representation of the primary sites and the percentage of metastasis per site).

Concerning anticancer treatment, 97.9% of the patients were receiving HTAs such as platinum-based agents, 5-FU, erythropoietin, or immunotherapy; more details are presented in Table 2. Furthermore, 26.5% of the patients had been surgically treated and 22.5% had a central venous catheter representing additional risk factors for thrombosis. Notably, a high percentage of these highly thrombogenic agents (71.5%) had potential drug–drug interactions (DDIs) with direct oral anticoagulants (DOACs). Agents with potential DDIs were identified according to bibliographical data [14–20] and are reported in the Appendix table 5.

**Table 2:** Cancer treatment agents and the percentage of cases that received such agents per primary cancer site

Primary site	Platinum-based compounds	Antimetabolites	Taxanes	Anti-VEGF agents	Plant alkaloid and topoisomerase I inhibitor	Immunotherapy agents	Erythropoietin agents
Breast	7.7%	42.3%	61.5%	42.3%	-	3.9%	3.9%
GI	67.6%	92.6%	34.3%	35.2%	31.5%	1.9%	30.6%
Head and neck	85.7%	71.4%	42.9%	0.0%	0.0%	57.1%	14.3%
Lung	65.2%	13.6%	48.5%	22.7%	4.6%	60.6%	28.8%
Urinary system	41.7%	33.3%	29.2%	33.3%	-	33.3%	37.5%
Woman reproductive system	85.1%	25.5%	70.2%	74.5%	2.1%	-	36.2%
Other	53.9%	46.2%	53.9%	38.5%	7.7%	30.8%	23.1%
% in all patients	60.7%	50.7%	45.3%	37.6%	13.1%	19.8%	27.9%

The average monitoring time from enlistment until last time a patient was seen was 148 ± 117 days. In terms of anticoagulation, in all patients was administered tinzaparin (10.000 Anti-Xa IU, OD) based on hospital protocol. The median anticoagulation duration for the study period was 6.2 months (Q1–Q3: 4.0–10.0 months). The duration was differentiated in the various cancer sites (*p* = 0.0621); Table 3 presents various characteristics of the patients, neoplasm details, anticancer treatment period, and anticoagulation approach in relation to cancer primary site. Notably, 15 patients with atrial fibrillation receiving oral anticoagulation switched to tinzaparin (10.000 Anti-Xa IU, OD) during the study period. In these patients, no thrombosis was reported, and 1 minor bleeding occurred.

**Table 3:** Anticoagulation approach in relation to cancer primary site, treatment, and patient

Neoplasm primary site	Incidence (%)	Age (> 65) (%)	Gender (F) (%)	Metastases (%)	HTAs (%)	Anticoagulation duration [median (Q1–Q3)]
GI	37.1	63.9	36.1	83	97	6.6 (4.8–9.8)
Lung	22.7	57.6	21.2	71.9	96.7	4.6 (2.2–7.5)
Woman reproductive system	16.2	51.1	100	54.4	100	6.5 (4.5–11.6)
Breast	8.9	57.7	100	65.4	83.3	7.5 (3.1–11.8)
Urinary system	8.3	70.8	12.5	82.6	87.5	6.1 (4–10.9)
Head and neck	2.4	14.3	28.6	50	100	7.6 (6–14.1)
Other	4.5	53.9	7.7	69.2	84.6	8.1 (4–16.3)

With regard to efficacy, six thrombotic events were observed (2.06%, 95% CI: 0.76–4.43%); from these, 5 events were DVT, and one was PE. Their major characteristics are presented in Table 4. All these patients had thrombosis history or suffered from cardiovascular disease or diabetes mellitus. However, the small number of thrombotic events is not sufficient to verify any relations of such events with the aspects recorded during MeTHOS study.

**Table 4:** Characteristics of the patients with thrombotic and bleeding events

	Age	ECOG PS	Gender	BMI	Ca primary site	Metastasis
Thrombotic events	37	0	M	26.3	Pancreas	Yes
	87	2	F	30.5	Colon	No
	71	0	F	29.4	Endometrium	Yes
	54	1	F	22.7	Endometrium, ovarian, tubal	No
	73	0	M	27.1	Gastric	Yes
	78	2	F	25.0	Breast	Yes
Bleeding events	51	1	M	26.3	Gastric	No
	66	0	M	26.3	Colon	Yes
	62	1	F	21.2	Pancreas	Yes
	62	0	M	26.3	Lung	Yes
	78	2	M	26.3	Pancreas	Yes
	76	1	M	26.3	Bladder	Yes
	68	0	F	18.7	Pancreas	Yes

With respect to safety, 7 bleeding events occurred (2.6%, 95% CI: 1.0–5.3%). Six of them were minor and one was major, in a 68-year-old woman with metastatic pancreatic cancer. BMI found to be related to bleeding (*p* = 0.0036), as the median BMI of the patients with bleeding was 20.0 (Q1–Q3: 18.7–25.4) while the patients that did not experienced bleeding had median BMI 26.0 (Q1–Q3: 23.0–29.4). Similarly, patients with lower body weight were more prone to bleeding (*p* = 0.0274); specifically, patients that experienced a bleeding event had median weight 58 kg (Q1–Q3: 53–75 kg) and the patients that did not experienced such events had median weight 71 kg (Q1–Q3: 60–83). As in the case of thrombotic events, the small number of bleeding events did not let to reveal any associations. Remarkably, 5 out of 7 bleeding events were related to the GI system and 5 out of 7 cases were men. However, no statistically significant difference can be confirmed, neither for the anatomical site nor for the gender.

## Discussion

In our hospital protocol were enrolled 291 active cancer patients with high thrombotic burden, which required thromboprophylaxis with low molecular weight heparin (LMWH) and the aim was to monitor efficacy and safety of thromboprophylaxis management in those patients. According to our results, the thromboprophylaxis management with tinzaparin 10,000 Anti-Xa IU, OD found to be effective without compromising the patients’ safety.

Cancer patients are fragile, usually of older age, have a poor performance status, with comorbidities requiring polypharmacy, high incidence of renal impairment, and are exposed to treatment combinations with potentially nephrotoxic effects [21]. Of note, in the current study, cancer patients had history of numerous simultaneous comorbidities, meaning almost 4 in 5 patients were dealt with polypharmacy, which is common in cancer [22] and also in thrombosis [23] patients. Furthermore, there is a close relationship between polypharmacy and DDIs. LMWH has been the recognized standard treatment for more than a decade, both in cancer-related thrombosis treatment and prevention [24]. Direct oral anticoagulants (DOACs) are a new option for anticoagulation therapy [25] but interactions with anticancer or other supportive drugs may be challenging, while for LMWHs, there are no known interactions. All DOACs are transported by P-glycoprotein, and in addition, rivaroxaban and apixaban are substrates for cytochrome P450 (CYP3A4) [26, 27]. Many drugs used in systematic anticancer therapy are inhibitors or inducers of P-glycoprotein and/or CYP3A4, which may potentially result in a change of plasma DOAC concentration, taking it outside the therapeutic window. The result of this may be lack of a therapeutic effect or an increase in the number of bleeding complications [28].

It is known that the thrombotic risk is highest for patients with certain malignancies, including lung, GI, and GU cancers. Lung cancer (LC) comprise the 22.7% of the current population and it is known that LC is a leading cause of cancer death in the USA for both men and women [29]. It is also one of the malignancies that are commonly associated with VTE, including PE, with reported incidence of VTE up to 13.8% and that of PE up to 3.8% [30–32]. Recently, Zhang et al. described the high prevalence of VTE in patients with newly diagnosed LC and VTE events occurred in 89 (13.2%) of the 673 patients enrolled in the study. Forty-two (6.2%) patients developed lower extremity deep vein thrombosis (DVT) alone, 33 (4.9%) patients developed pulmonary embolism (PE), and 14 (2.1%) patients developed both DVT and PE [33]. No VTE events were reported in lung cancer patients in our cohort.

High rates of symptomatic and incidental thromboembolic events have been reported in gastrointestinal (GI) cancer patients which represent the 37.1% in the existing cohort. In a retrospective study which included a total of 220 consecutive GI cancer patients, sixty patients (27.3%) were found to have experienced a total of 83 thromboembolic events. These included 32 DVTs (38.6%) and 17 PEs (20.5%). An additional twenty three patients developed 25 (30.1%) visceral vein or 9 (10.8%) arterial thromboembolic events [34]. Only one VTE event reported in GI cancer patients in our analysis, specifically in a pancreatic cancer patient. In the present population, the 33% of the GI malignancies were patients with pancreatic cancer. Reported frequencies of thrombosis associated with pancreatic cancer are the highest compared with other malignancies. The first case series describing the striking relationship between pancreatic cancer and thrombosis was published in 1938 where it is documented a 60% prevalence of venous thromboembolism in patients with pancreatic cancer at autopsy. Studies carried out over the past 10–15 years have reported VTE prevalence rates of up to 36% in patients with pancreatic cancer [27].

Gynecologic cancer patients constituted 16.2% of the current study population and this cancer type has been also associated with high incidence of VTE in previous trials. In a retrospective study, among 1885 women with gynecologic cancer, 769 (40.8%) experienced venous thromboembolic events, most of them in the first 2 years after cancer diagnosis. Specifically, 40.4% of patients experienced DVT, while PE occurred in 1.2% of the patients. There was no statistically significant difference in the incidence of VTE according to the type of gynecologic cancer [35]. Two VTE events were reported in patients with gynecologic cancer in our cohort.

The presence of metastases is associated with increased hypercoagulability, as the hemostatic system seems to play a key role in the metastatic capacity of solid tumors [36]. Additionally, patients with metastatic disease at the time of diagnosis had up to 21.5 times higher risk for thromboembolism in comparison to patients with non-metastatic disease. Also, there is data shown that mucinous adenocarcinomas, such as pancreatic, lung, and cancers of the gastrointestinal tract, are those with the highest incidence of cancer-related VTE [37–39]. Cancer patients with metastatic disease comprised the 72.9% of the current study population and metastasis is considered a dominant factor for VTE. Four VTE events and two bleedings were observed in metastatic patients in our analysis. In a similar cancer population, VTE prevalence was found to increase with stage sharply in patients with tumors at a higher stage [40, 41]. Similarly, advanced disease stages and distant metastases increase VTE risk as it is shown in the Blom et al. report where an adjusted odds ratio (OR) of 19.8 for VTE risk was noticed in patients with metastatic cancer compared with patients without overt metastases [42].

Cancer therapy and thrombosis for over three decades remained an underappreciated risk that has not been routinely incorporated into thrombosis risk assessment models [43]. Mechanisms that drive cancer therapy–associated thrombosis are not fully understood. A primary mechanism may be the activation or disruption of the endothelium by anticancer agents. In addition, these agents may decrease anticoagulants and increase procoagulants, such as tissue factor (TF), leading to activation of coagulation. Finally, anticancer drugs may directly or indirectly activate platelets [44].Cancer patients undergoing systemic treatment for their malignancy are among the highest risk populations for thromboembolic complications; highly thrombogenic chemotherapy agents (HTAs) include platinum compounds, 5-FU, capecitabine, gemcitabine, hormonal therapy, anti-angiogenesis treatment, e.g., bevacizumab, and supportive treatment, e.g., corticosteroids, erythropoietin [45]. VTE is also common in cancer patients receiving immunotherapy either as single-agent or combination regimens, affecting nearly one-third of all immunotherapy treated patients and may potentially be associated with worsened survival [46]. HTAs were used at 97.9% the present population.

In a retrospective observational study [47] of 18,531 patients diagnosed with a malignant tumor, the majority of VTE events occurred shortly after the diagnosis of cancer. Among the cancer patients, 53.92% had a VTE event within the first 3 months, 63.21% within the first 6 months, and 68.93% within the first 9 months (notably patients experienced more than a single VTE event). The median duration of anticoagulation treatment during the present study period was 6.2 months representing also the median duration of antineoplastic treatment.

The LMWHs constitute standard of care along with DOACs for the treatment and prevention of VTE for patients with active cancer, without the warning for the safety considerations and DDIs that follow the DOACs [48, 49]. There is strong evidence that the coagulation plays an important role in cancer metastasis and angiogenesis [50]. The anti-tumor effect of heparins, particularly LMWH, has been confirmed. These anticoagulants inhibit cancer cell growth and metastasis formation in several ways. The anti-angiogenic effect of LMWH is found to be expressed in a dose-dependent manner [51, 52]. Tinzaparin sodium Xa inhibitory effect is dose-dependent, and higher, as compared to its anti-IIa activity [53]; moreover, it disposes the highest anti-IIa activity among all LMWHs. Since LMWHs express “pleiotropic effects” in a dose-dependent manner, a “high thrombotic burden (HTB)”-adapted strategy could help high-risk patients who may benefit beyond anticoagulation from use of higher than conventional prophylactic LMWH dose. Notably, in recently published data, intermediate-dose tinzaparin (8000–12,000 Anti‑Xa IU, once daily) was found to be more efficacious for the prevention of VTE, without compromising safety [54]. Tinzaparin sodium possesses important pharmacokinetic properties, with the consecutive involvement of cellular and renal route of elimination, exhibiting no bioaccumulation even in patients with severe renal impairment, maintaining a special stand among other LMWHs [55] [56]. With the above mentioned characteristics, tinzaparin seems that reconciles the relevant profile for patients with active cancer who combine multiple factors worsening their renal function. Such factors include but are not limited to: older age, dehydration, use of nephrotoxic agents for anticancer treatment and other comorbidities, such as hypertension and diabetes mellitus [55]. Furthermore, anticancer effects have also been delineated in vitro, in vivo, and retrospective trials [57–60].

The effect of thromboprophylaxis with other LMWHs in various solid tumors is considered in two main RCTs: SAVE ONCO [61] using semuloparin and PROTECT [62] using nadroparin. Patients’ characteristics and malignancies are comparable with current cohort. In PROTECT, the median prophylaxis duration was less than 4 months, and in SAVE ONCO, it was 3.5 months, while in our cohort, the average duration was longer than 6 months. With regard to efficacy, thromboembolic events were experienced by 2.0% of the patients treated with nadroparin in PROTECT and by 1.2% of the patients receiving semuloparin in SAVE ONCO similar to our study (2.06%).

In terms of safety, minor bleeding events occurred in 7.4% of patients treated with nadroparin in the PROTECT study, and major ones in 0.7% of them. The incidence of clinically relevant bleeding in SAVE ONCO was 2.8%, and that of major bleeding 1.2% in the semuloparin group. In the MeTHOS cohort, 7 bleeding events occurred (2.6%). Six of them were minor and one major. In both PROTECT and SAVE ONCO trials, the dose used was the prophylactic one.

Our study had the limitations and advantages of a pragmatic study [63] designed in a broad range of routine clinical practice, without specific focus on patients’ characteristics; thus, unknown bias could have been introduced. There was no selection of patients into intervention. Therefore, in the authors’ opinion, this study captured the real-life conditions in a routine clinical oncology setting. One of the strengths of our approach was the validity of our results, related to efficacy and safety due to the fact that thromboprophylaxis duration lasted 6 months.

ffffffffThe risk of VTE is increasing in patients with active cancer and the MeTHOS study demonstrates that it is important to assess the thrombotic burden in patients receiving anticancer treatment. Individuals at increased thrombotic risk should be offered thromboprophylaxis to avoid serious and life-threatening complications. The administration of LMWH (tinzaparin intermediate dose 10,000 Anti-Xa IU, OD) appears to offer an effecctive and safe solution for thrombo-prophylaxis during the course of anti-cancer treatment.

## Data Availability

Study data are available from the corresponding author upon reasonable request.

## Appendix

**Table 5:** Potential drug–drug interactions according to bibliographic information [14–20]

Treatment	Risk of bleeding	Pharmacokinetic implications	GI implications	Hematological implications
Abemaciclib	No	-	X (D)	A, T
Abiraterone	No	-	X (D)	-
Afatinib	No	-	X (D)	Epistaxis
Alectinib	No	CYP3A4_s_, P-gp_inh_	X (D)	-
Anastrozole	Yes	-	X (D)	-
Bevacizumab	Yes	-	X (D, S)	A, T
Brigatinib	No	CYP3A4_s_, CYP3A4_ind_, P-gp_s_, P-gp_inh_	X (D)	-
Cabazitaxel	Yes	-	X (D, S)	A, T
Capecitabine	Yes	-	X (D, S)	A
Carboplatin	Yes	-	D	A, T
Ceritinib	No	CYP3A4_s_, CYP3A4_ind_, P-gp_s_	X (D)	A
Cetuximab	No	-	D, M	-
Cisplatin	No	-	S	A, T
Continuous infusion -FU	No	-	X (D, S)	A, T
Crizotinib	No	CYP3A4_s_, P-gp_inh_	X (D)	A
Cyclophosphamide	Yes	CYP3A4_s_	X	-
Dabrafenib	No	CYP3A4_s_, CYP3A4_ind_, P-gp_s_	X (D)	A,T
Dacomitinib	No	P-gp_s_	X (D)	-
Docetaxel	Yes	CYP3A4_s_	X (D, S)	A, T
Doxorubicin	Yes	P-gp_s_, P-gp_ind_, CYP3A4_s_	D, S	A, T
Entrectinib	No	CYP3A4_s_, P-gp_s_	X (D)	A
Enzalutamine	No	CYP3A4_ind_	-	-
Epirubicin	No	-	X (D, S)	A, T
Eribulin	No	-	X (D, S)	A, T
Erlotinib	No	CYP3A4_s_	X (D)	Epistaxis, GI Bleeding
Etoposide	No	-	D, S, M	A, T
Exemestane	No	CYP3A4_s_	X (D)	T
Fluorouracil	No	-	X (D, S)	A, T
FOLFOX (Preferred)	Yes	-	D, S, M	A, T
Fulvestrant	Yes	-	X (D)	-
Gefitinib	No	CYP3A4_s_	X (D)	Epistaxis and haematuria
Gemcitabine	No	-	X (D, S)	A, T
Irinotecan	No	CYP3A4_s_	X (D)	A, T
Larotrectinib	No	CYP3A4_s_, P-gp_s_	X	A
Letrozole	Yes	-	X (D)	-
Lorlatinib	No	CYP3A4_s_, P-gp_inh_	X (D)	A
Megestrol acetate	No	-	X (D)	-
Mitoxantrone	No	-	X (D, S)	A, T
Niraparib	Yes	P-gp_s_, CYP3A4_s_	D, S	A, T
Nivolumab	No	-	D, C, S	A, T
Olaparib	No	CYP3A4_s_	D, S	A, T
Osimertinib (preferred)	No	-	X (D)	Platelet count decreased
Paclitaxel	Yes	P-gp_s_, CYP3A4_s_	X (D, S)	A, T
Panitumumab	Yes	-	D, S	A
Pembrolizumab	No	-	X (D, S)	A, T
Radium-	No	-	X (D)	T
Ramucirumab	Yes	-	X (D, S)	A, T
Rucaparib	Yes	P-gp_s_	D	A, T
Tamoxifen	Yes	CYP3A4_s_	X (D)	A
Topotecan	No	P-gp_s_	D, M	A, T
Toremifene	Yes	-	X	-
Trametinib	Yes	P-gp_s_	X (D)	A,T
Trifluridine	No	-	X (D, S)	A, T
Vinorelbine	No	P-gp_s_, CYP3A4_s_	X (D, S)	A, T

GI implications–X (e.g., nausea/vomiting colitis/diarrhea/mucositis); *D*, diarrhea; *A*, anemia; *T*, thrombocytopenia; *S*, stomatitis; *M*, mucositis substrate of CYP3A4 (CYP3A4s); inhibitor of CYP3A4 (CYP3A4inh); inducer of CYP3A4 (CYP3A4ind); P-gp inhibitor (P-gpinh); P-gp inducer (P-gpInd); P-gp substrate (P-gps). Only strong inhibitors and inducers are noted. Common or very common adverse events were included. The clinical relevance of the pharmacokinetic implications is not known
